# Protein Kinase D2 Is an Essential Regulator of Murine Myoblast Differentiation

**DOI:** 10.1371/journal.pone.0014599

**Published:** 2011-01-27

**Authors:** Alexander Kleger, Christiane Loebnitz, Ganesh V. Pusapati, Milena Armacki, Martin Müller, Stefan Tümpel, Anett Illing, Daniel Hartmann, Cornelia Brunner, Stefan Liebau, Karl L. Rudolph, Guido Adler, Thomas Seufferlein

**Affiliations:** 1 Department of Internal Medicine I, University of Ulm, Ulm, Germany; 2 Institute of Molecular Medicine and Max-Planck-Research Department on Stem Cell Aging, University of Ulm, Ulm, Germany; 3 Department of Surgery, Technical University Munich, Munich, Germany; 4 Institute of Physiological Chemistry, University of Ulm, Ulm, Germany; 5 Institute of Anatomy and Cell Biology, University of Ulm, Ulm, Germany; 6 Department of Internal Medicine I, Martin Luther University Halle-Wittenberg, Halle, Germany; Ohio State University, United States of America

## Abstract

Muscle differentiation is a highly conserved process that occurs through the activation of quiescent satellite cells whose progeny proliferate, differentiate, and fuse to generate new myofibers. A defined pattern of myogenic transcription factors is orchestrated during this process and is regulated via distinct signaling cascades involving various intracellular signaling pathways, including members of the protein kinase C (PKC) family. The protein kinase D (PKD) isoenzymes PKD1, -2, and -3, are prominent downstream targets of PKCs and phospholipase D in various biological systems including mouse and could hence play a role in muscle differentiation. In the present study, we used a mouse myoblast cell line (C2C12) as an *in vitro* model to investigate the role of PKDs, in particular PKD2, in muscle stem cell differentiation. We show that C2C12 cells express all PKD isoforms with PKD2 being highly expressed. Furthermore, we demonstrate that PKD2 is specifically phosphorylated/activated during the initiation of mouse myoblast differentiation. Selective inhibition of PKCs or PKDs by pharmacological inhibitors blocked myotube formation. Depletion of PKD2 by shRNAs resulted in a marked inhibition of myoblast cell fusion. PKD2-depleted cells exhibit impaired regulation of muscle development-associated genes while the proliferative capacity remains unaltered. Vice versa forced expression of PKD2 increases myoblast differentiation. These findings were confirmed in primary mouse satellite cells where myotube fusion was also decreased upon inhibition of PKDs. Active PKD2 induced transcriptional activation of myocyte enhancer factor 2D and repression of Pax3 transcriptional activity. In conclusion, we identify PKDs, in particular PKD2, as a major mediator of muscle cell differentiation *in vitro* and thereby as a potential novel target for the modulation of muscle regeneration.

## Introduction

The process of skeletal muscle differentiation is characterized by mononucleated myoblasts exiting cell cycle and fusing to form multinucleated myotubes. This process is orchestrated at the molecular level by the expression of the myogenic basic helix-loop-helix (bHLH) transcription factors MyoD, Myf5, myogenin, and MRF4[1]. Myogenic bHLH factors cooperate with each other and with the myocyte enhancer factor-2 (MEF-2) family of transcription factors to regulate their own expression in a feedback loop that maintains the transcriptional program [2], [3], [4], [5]. This results in expression of myofibrillar proteins, such as myosin heavy chain (MHC)[6]. In addition to myogenic transcription factors, several protein kinases including p38-MAPK [7], PLD [8] and PKC isoforms [9], [10] have been implicated in the regulation of skeletal muscle stem cell differentiation.

The protein kinase D (PKD) family belongs to the calcium-/calmodulin-dependent protein kinase superfamily [11] and comprises the three isoforms, PKD1, -2 and -3 [12]. PKDs are activated by various stimuli, including phorbol esters, G-protein-coupled receptors and reactive oxygen species [12], [13]. PKDs are serine threonine kinases and act as prominent downstream targets of PKCs, including novel PKCη and ε [14], [15]. PKCs directly activate PKDs via phosphorylation at two critical serine residues within the activation loop of the catalytic domain [16]. However, PKDs can also be activated by direct binding of diacylglycerol (DAG) to the C1a domain within their regulatory domain [17]. The members of this family play a role in cell motility, migration, and invasion. Furthermore, they regulate protein transport by inducing the fission of budding vesicles from the *trans*-Golgi network [18], [19], [20], [21]. Interestingly, members of the PKD family were found to play a role in skeletal, cardiac, and smooth muscle regulative processes: PKD1 stimulates MEF2 activity in skeletal muscle, enhances muscle performance and plays a crucial role in pathological heart remodeling [22], [23]. In addition, the hypertrophic response to angiotensin II in smooth muscle cells is mediated by PKDs [24].

Development and regeneration of the skeletal muscle is largely dependent on a small population of resident cells termed satellite cells which are capable of terminal differentiation during these processes. Here, we examined a potential role of PKDs during myogenic differentiation using murine C2C12 myoblasts to recapitulate myogenic differentiation. These cells differentiate rapidly, form contractile myotubes, express characteristic marker genes and, therefore, serve as a bona fide *in vitro* model to study the differentiation of myoblasts to myotubes [25].

We show that C2C12 cells predominantly express PKD2 and PKD3. PKD2 was found to be the major PKD isoform that was catalytically active during the early phase of differentiation of C2C12 cells to skeletal muscle cells. Pharmacological inhibition of PKCs or PKDs led to a markedly decreased differentiation of C2C12 cells. Ectopic expression of active PKD2 induced transcriptional activation of Mef2D and inhibited transcriptional activity of Pax3 in these cells. Furthermore, selective depletion of PKD2 by specific shRNAs was sufficient to inhibit/prevent differentiation of C2C12 cells. Vice versa overexpressed PKD2 induced differentiation. Collectively, our data demonstrate an essential role of PKD2 in the differentiation of precursor cells to skeletal muscle cells potentially leading to novel treatment strategies for *in vivo* muscle regeneration.

## Results

### Expression of PKDs in C2C12 cells and adult skeletal muscle

**Figure 1:**
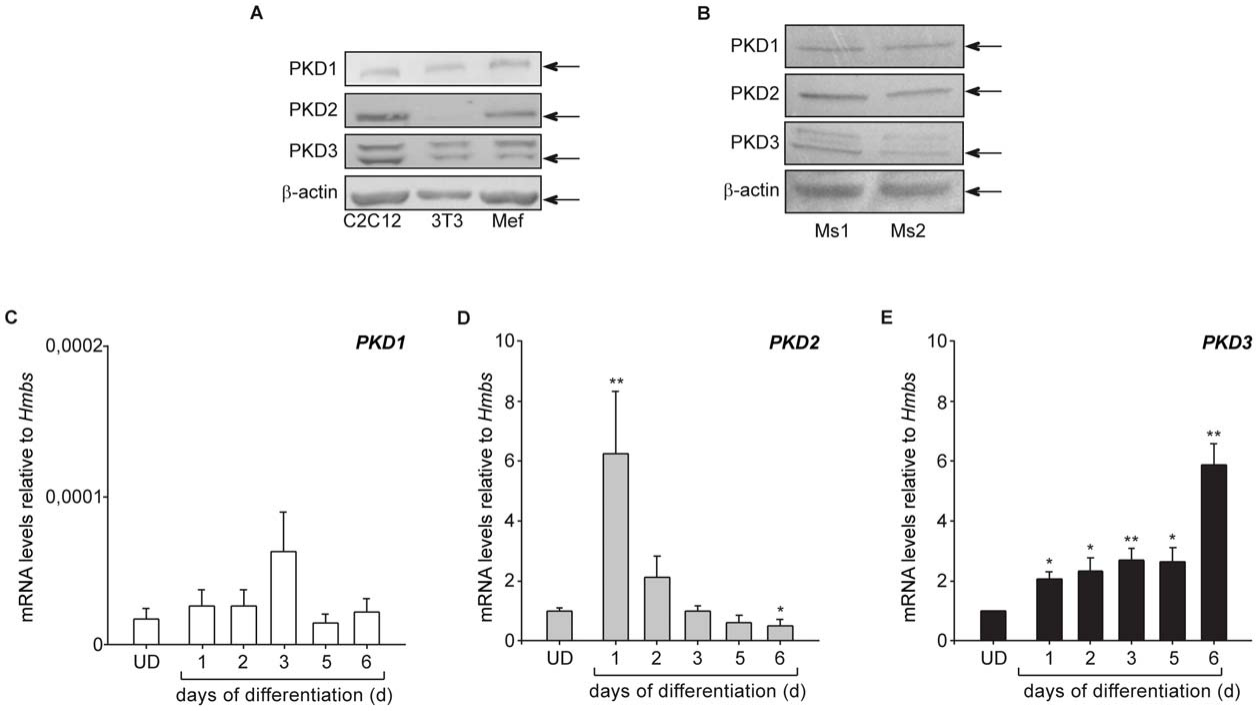
PKD2 is the predominant isoform in undifferentiated and in differentiated C2C12 cells. (**A**) Immunoblot analysis for PKD1, 2, and 3 in undifferentiated C2C12 cells. Murine embryonic fibroblasts and NIH3T3 fibroblasts served as controls. One representative blot from three independent experiments is shown. (**B**) Immunoblot analysis for PKD1, 2, and 3 in terminally differentiated murine skeletal muscle. Lysates of the right and left tibial anterior muscle of two mice are shown. (**C–E**) qPCR analysis of PKD1 (**C**), PKD2 (**D**), and PKD3 (**E**) expression over time in C2C12 cells and their differentiated progeny at the indicated time points. Relative gene expression was determined as a ratio of target gene concentration to the concentration of a housekeeping gene, murine hydroxymethylbilane synthase (mHMBS). Three independent experiments ±SEM were tested for significance using the Student's t-test. P<0.05 (*) and p<0.01 (^**^) are indicated.

PKDs are expressed in the developing mouse embryo. In particular PKD2 shows a differential regulated expression pattern and is highly abundant in the heart and striated muscles while PKD3 lacks striated muscle expression [26]. Additionally PKDs play a major role in terminally differentiated muscle types. Therefore, we investigated the expression of PKD isoforms in the mouse myoblast cell line C2C12 in order to assess whether PKDs could be important for myogenic stem cell differentiation. As shown in **Figure 1A**, PKD2 and PKD3 were highly expressed in C2C12 cells and appeared as the predominant PKD isoforms of this particular cell type. PKD1 was found to be expressed at very low levels compared to the other isoforms in C2C12 cells at both, the protein and the mRNA level (**Figure 1A and 1C**). In terminally differentiated murine skeletal muscles, PKD2 was found to be the predominant isoform (**Figure 1B**).

Next, we examined the expression of the various PKD isoforms during myoblast differentiation in the C2C12 cell system. As shown in **Figure 1C**, there was very little PKD1 expression at baseline. In addition, PKD1-mRNA did not change substantially during differentiation (**Figure 1C**). In contrast, there was an immediate, 6-fold increase in PKD2 mRNA at day 1 after initiation of differentiation whereas PKD2 mRNA levels were markedly lower during the terminal myotube stages (**Figure 1D**). Interestingly, PKD3 mRNA expression exhibited different kinetics during differentiation of C2C12 cells: PKD3 mRNA was only slightly induced upon initiation of differentiation and during mid stage differentiation, but peaked at day 6 of myotube differentiation (**Figure 1E**). These data suggest that PKD2 could be the major isoform involved in the early stages of myogenic differentiation.

### PKD2 is activated by sphingosine-1-phosphate (S1P) in C2C12 cells

**Figure 2:**
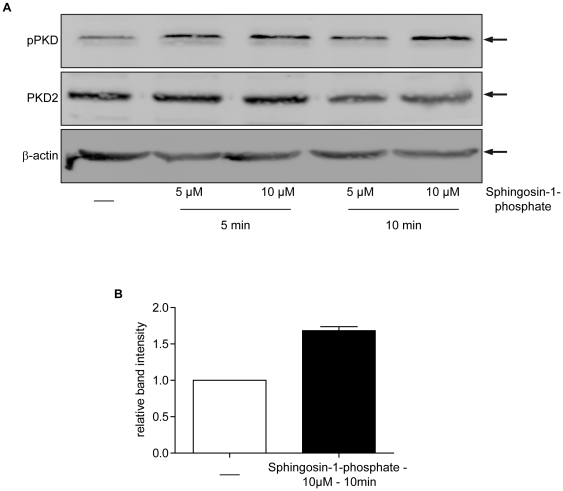
PKD2 is activated by sphingosine-1-phosphate (S1P) in C2C12 cells. Subconfluent C2C12 cells were stimulated as indicated in the figure with (S1P) and further processed for immunoblot analysis for PKD2 and phospho-PKD (Ser 744/748). (**A**) One representative western blot for PKD2 and phospho-PKD (Ser 744/748) out of three independent experiments is shown. β-actin served as loading control. (**B**) Quantification of band intensity in the shown immunoblot of phospho-PKD expression relative to PKD2 in S1P-stimulated C2C12 cells. Values include 10 min values of 10 µM S1P treatment from all three experiments.

Next, we were interested whether agents known to induce myogenic differentiation were able to activate PKDs. Insulin like growth factors (IGFs) are major mediators of myogenic stem cell differentiation [27], [28], [29] and have been described to activate PKDs [30]. However, in contrast to myeloma cells, incubation of undifferentiated C2C12 cells with different concentrations of IGF-1 did not result in activation of PKD2 (data not shown). Another ligand known to be involved in myogenic differentiation is the bioactive lipid S1P that acts on a heptahelical receptors [31]. S1P acts as a powerful pro-differentiating agent by enhancing the expression of myogenic differentiation markers such as myogenin, myosin heavy chain, and caveolin-3 [31], [32], [33]. As shown in **Figure 2A and 2B**, S1P markedly stimulated PKD phosphorylation/activation. Thus, PKDs can be activated by a known stimulator (S1P) of myogenic differentiation in C2C12 cells.

### PKD2 is activated during the initiation of myoblast differentiation

**Figure 3:**
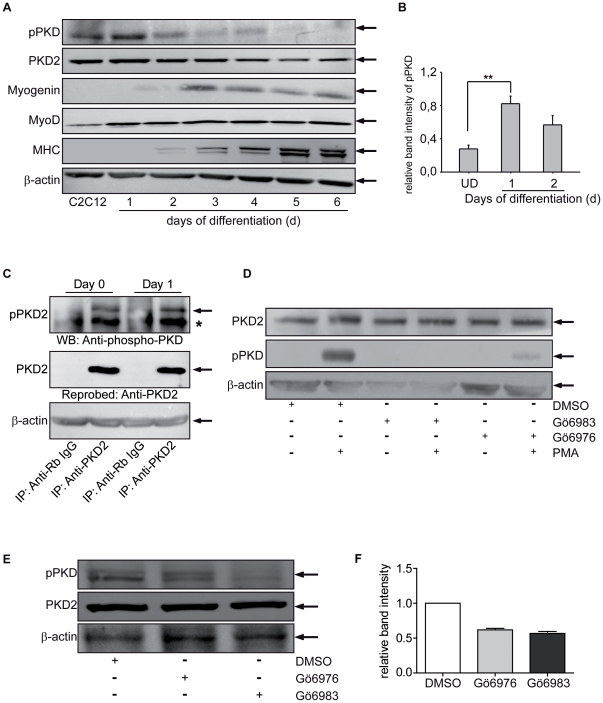
PKD2 is activated during muscle stem cell differentiation. (**A**) Immunoblot analysis for PKD2 and phospho-PKD (Ser 744/748) during *in vitro* differentiation of C2C12 cells at the indicated time points. C2C12 cells express the expected marker profile after induction of differentiation. Time points are as indicated in the figure. Upper immunoblot shows myogenin followed by MyoD and MHC, respectively. β-actin served as the corresponding loading control. One representative experiment out of three is shown. (**B**) Quantification of band intensity of phospho-PKD expression normalized to PKD2 levels during C2C12 cell differentiation is shown at day 0, 1, and 2. (**C**) Immunoprecipitation analysis for PKD2 followed by immunoblot for phospho-PKD (Ser 744/748) during *in vitro* differentiation of C2C12 cells at the indicated time points. One representative experiment out of three is shown. (**D**) Undifferentiated, not confluent C2C12 cells were treated for one hour with either 5 µM Gö6976 or Gö6983: DMSO served as a solvent control. As indicated, cells were additionally treated with PMA for 10 minutes. After compound treatment, cells were harvested and subsequently analyzed by immunoblotting using PKD2 or phospho-PKD (Ser 744/748) antibodies. Exposure time was very short in purpose to avoid overexposure and interference with neighboured bands. Shown is one representative experiment out of two independent experiments with similar results. (**E**) C2C12 cells were treated one day prior to induction of differentiation by serum withdrawal with 5 µM Gö6976 and 5 µM Gö6983. On day 0, cells were switched to low serum conditions. DMSO served as a solvent control. On day 1, cells were lysed and subsequently analyzed by immunoblotting using PKD2 or phospho-PKD (Ser 744/748) antibodies. Shown is one representative experiment out of three independent experiments. (**F**) Quantification of band intensity of phospho-PKD expression normalized to PKD2 levels is shown during C2C12 cell differentiation at day 1.

Since PKD2 was found be the major isoform expressed during the initial stages of differentiation at the level of gene expression, we focussed on the role of this isoform in the process of muscle stem cell differentiation. To examine the role of PKD2 in myogenesis, we assessed PKD2 expression and activity in C2C12 cells upon differentiation. C2C12 cells grown to 90% confluence were induced to differentiate by serum withdrawal. Typically, cell fusion and formation of small myotubes were evident 3 days after induction and myotubes were fully formed within 5-6 days (**Figure S1**). PKD2 protein expression correlated with mRNA expression profiles and slowly decreased during differentiation (**Figure 3A**). The PKD2 antibody used in this study was specific in detecting only PKD2 and did not show cross reactivity with either PKD1 or PKD3 (**Figure S2**). Undifferentiated C2C12 cells grown to confluency also exhibited a substantial basal PKD activity as demonstrated by PKD phosphorylation within the activation loop at two conserved serine residues that indicate catalytic activity (**Figure 3A**) [15]. Activation loop phosphorylation and therefore PKD catalytic activity were significantly further increased upon initiation of differentiation by serum withdrawal (two-fold increase, **Figure 3B**). Our hypothesis that PKD2 is the major isoform that is activated in the muscle stem cell differentiation process was further substantiated by immunoprecipitation of PKD2 followed by detection of PKD2 activation using the phospho-specific PKD antibody (**Figure 3C**). PKD2 activity was increased on day 1 of differentiation.

PKD2 activation and peak protein expression were followed by increased myogenin and MyoD expression; both are key players in the initiation process of myogenesis. As a control for the differentiation process in our cell culture system, we determined the expression of MHC that gradually increased as expected until day 5 (**Figure 3A**). Hence, PKD phosphorylation coincides with both critical initiation steps of myoblast differentiation, namely cell density and serum starvation. (**Figure 3A–C**).

Activation loop phosphorylation and hence catalytic activity of PKD could be further increased upon incubation of subconfluent, undifferentiated C2C12 cells with the phorbol ester PMA, a potent activator of both PKCs and PKDs (**Figure 3D**) [12]. PKCs are major regulators of PKD activity in various cell systems [15]. Gö6983 inhibits predominantly the catalytic activity of classical and novel PKCs η,θ,δ,ε whereas Gö6976 selectively inhibits the classical PKCα and PKDs [34]. Incubation of cells with Gö6983 (5 µM) completely abrogated basal and PMA-stimulated activation of PKD demonstrating that PKCs are major regulators of PKDs also in C2C12 cells (**Figure 3D**). The selective PKD inhibitor Gö6976 (5 µM) also inhibited PKD phosphorylation in subconfluent C2C12 cells. The effect of both inhibitors on PMA-induced PKD2 phosphorylation was concentration-dependent with a maximum inhibitory effect observed between 5 and 10 µM (data not shown). To examine whether these inhibitors could also abolish differentiation-induced PKD phosphorylation, we treated confluent C2C12 cells with the respective inhibitors after serum withdrawal. PKD phosphorylation in response to serum withdrawal was blocked by both inhibitors during differentiation (**Figure 3E and 3F**). Thus, PKDs are constitutively phosphorylated within the activation loop in subconfluent, undifferentiated C2C12 cells and activation of PKDs is mediated by PKCs in these cells. Activation of PKDs in response to differentiation-inducing agents can be blocked by Gö6983 and Gö6976.

### Pharmacological inhibition of PKCs and PKDs inhibits myoblast differentiation *in vitro*

**Figure 4:**
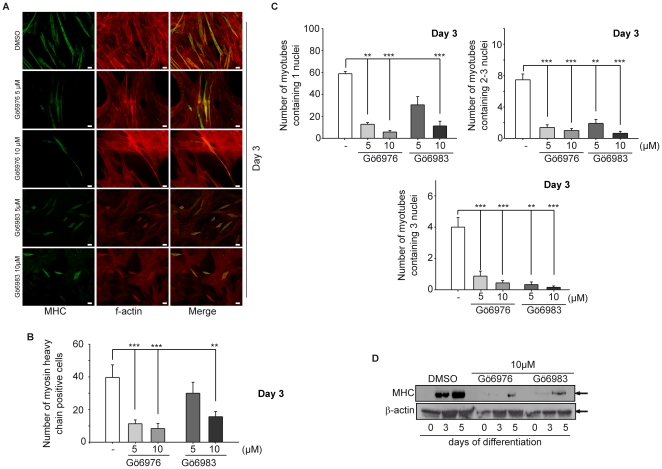
Pharmacological inhibition of PKCs and PKDs inhibits myoblast differentiation *in vitro*. (**A**) Cells were seeded on cover slips and treated with either Gö6976 or Gö6983. Concentrations are indicated in the figure. DMSO served as a solvent control. On day 3, cover slips were stained for MHC (green), F-actin (red) and nuclei (blue). Photographs are representative for 3 independent experiments. Scale bars, 20 µM. (**B**) Number of myotubes or MHC-positive cells per visual field. Ten randomly selected visual fields were photographed and the number of MHC positive cells was plotted as indicated in the figure. P<0.05 (*), p<0.01 (^**^) and p<0,001 (***). (**C**) Fusion indices for inhibitor (Gö6076 and Gö6983) treated cultures in comparison to solvent controls are shown. Concentrations are indicated in the figure. Fusion indices were calculated as the number of nuclei per myotube and sub-classified as 1 nucleus per myotube (upper left panel), 2-3 nuclei per myotube (upper right panel) or more than 3 nuclei per myotube (lower panel). Ten randomly selected visual fields were evaluated. P<0.05 (*), p<0.01 (^**^) and p<0.001 (***). (**D**) Immunoblot analysis for MHC in differentiating C2C12 cells. Cultures were treated with inhibitors (Gö6076 and Gö6983) as indicated. One representative immunoblot out of two is shown.

Next, we were interested whether PKD2 activity is required for myogenesis. C2C12 cells were differentiated for 5 days by serum withdrawal in the presence of Gö6983 or Gö6976 to inhibit PKC/PKD activity. As shown in **Figure 4A**, treatment with Gö6983 or Gö6976 reduced the degree of C2C12 differentiation as evidenced by visualizing MHC-positive myotubes. DAPI staining was performed to determine cell density and calculation of the fusion index (channel not shown). F-actin staining was used to visualize the morphology of all cell types. The solvent control did not exhibit any effect on differentiation. Inhibitor treatment markedly reduced the total number of myotubes per visual field at day 3 (**Figure 4A and 4B**) and day 5 (**Figure S3A** and **S3B**). This suggests an effect of the inhibitors already during the initiation of differentiation as MHC exclusively stains differentiated myoblasts already expressing myofibrillar proteins. To assess the differential effects of the inhibitors more precisely, we calculated not only the number of myotubes per visual field but also the fusion index to assess the relative differentiation potential. Separate treatment of cells with both inhibitors dramatically reduced the fusion index in all groups (**Figure 4C** and **Figure S3C**). At 10 µM Gö6976 induced cell death. Gö6983 used at the same concentration induced less cell death (data not shown), but was also less potent in reducing the fusion index compared to Gö6976 (**Figure 4B and 4C**). Notably, the morphology of myotubes differed in both inhibitor-treated groups. Gö6983-treated myotubes displayed a significantly decreased length-to-width ratio while in Gö6976-treated myotubes the length-to-width ratio was increased compared to control cells both at day 3 and day 5 (**Figure S4**). Given that Gö6983 predominantly inhibits PKCs and Gö6976 PKDs, these data suggest that PKDs may have additional roles in myoblast differentiation apart from acting as PKC downstream targets. Both, Gö6976 and Gö6983 significantly reduced the expression of the contractile protein MHC in C2C12 cells (**Figure 4D**).

### shRNA-mediated PKD2 depletion inhibits myoblast differentiation *in vitro*

**Figure 5:**
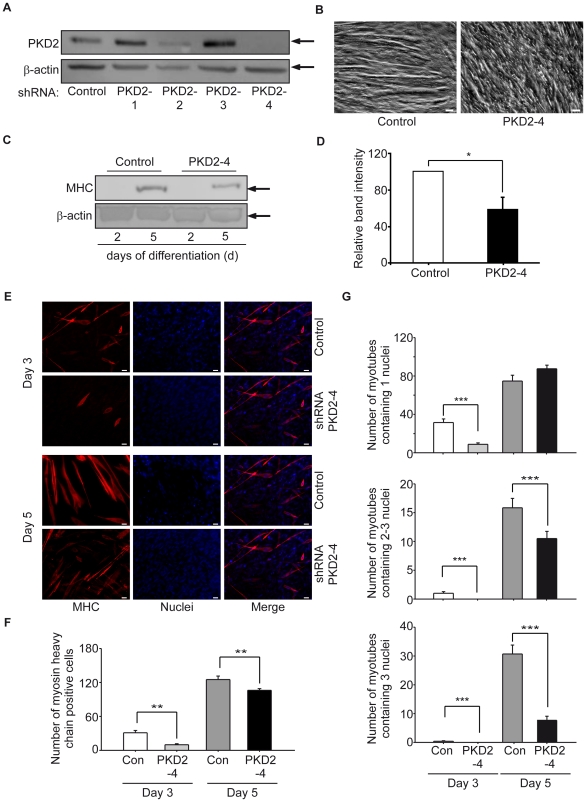
shRNA-mediated PKD2 depletion inhibits myogenesis *in vitro*. (**A**) C2C12 cells were infected with lentiviruses encoding for different shRNAs targeting PKD2 according to *material and methods*. After infection cells were selected for one week with puromycin to generate stable cell lines. One representative immunoblot for PKD2 in different cells generated with the indicated shRNA lentivirus is shown. (**B**) Representative bright field view of control virus transduced C2C12 cells (left photograph, control) and shRNA #4 targeting PKD2 (right photograph, PKD2-4) are shown. Scale bars, 20 µM. (**C**) Control cells (Control) and shRNA#4 against PKD2-transduced cells (PKD2-4) were differentiated and collected as indicated in the figure. Subsequent immunoblot analysis revealed diminished MHC expression in knock down cells. One representative immunoblot is shown. (**D**) Quantification of PKD2 immunoblots from (**C**) in three independent experiments is shown. P<0.05 (*) and p<0.01 (^**^). (**E**) Control virus (Control) infected cells and PKD2 knock down cells (PKD2-4) were seeded on cover slips. On day 3 and 5, cover slips were stained for MHC (green) and nuclei (blue). Photographs are representative for three independent experiments. Scale bars, 20 µM. (**F**) Number of myotubes or MHC-positive cells per visual field. Ten randomly selected visual fields were photographed and number of MHC positive cells were plotted as indicated in the figure. Control cells (Con). PKD2 knock down cells (PKD2-4). Wilcoxon statistical test was used. P<0.05 (*), p<0.01 (^**^). (**G**) Fusion indices of both stable cell lines are shown. Control cells (Con). PKD2 knock down cells (PKD2-4). €Fusion indices were calculated as the number of nuclei per myotube and sub-classified as 1 nucleus per myotube (upper left panel), 2-3 nuclei per myotube (upper right panel) or more than 3 nuclei per myotube (lower panel). Ten randomly selected visual fields were evaluated. P<0.05 (*), p<0.01 (^**^) and p<0.001 (***).

To specifically address the role of PKD2 during myoblast differentiation, we depleted PKD2 in C2C12 cells by lentiviral mediated shRNAs targeting specifically PKD2. Initially, stable cell lines with four independent shRNAs targeting the PKD2 gene were generated. A cell line carrying the pLKO vector with GFP served as a negative control (Control, Con). shRNAs targeting PKD2 specifically reduced the amount of PKD2 protein by either 50% (#PKD2-2) or 90% (#PKD2-4). PKD1 and -3 protein levels were not altered by the PKD2 shRNA (data not shown). The shRNA constructs #PKD2-1 and 3 did not have a significant effect on PKD2 expression (**Figure 5A**). Upon depletion of PKD2, we observed an altered morphology and a reduced number of myotubes (**Figure 5B**). Gene expression analysis during differentiation revealed strongly altered expression profiles in both PKD2 depleted cell lines (#PKD2-2, #PKD2-4; **Figure S5A-E**). The activation of satellite cells is associated with an initial simultaneous stimulation of Pax7 and MyoD expression. The downregulation of Pax7 by constant MyoD expression is described to be the initial signal for differentiation of the cells (**Figure S5A–B**) [35], [36], [37], [38]. When Pax 7 is down-regulated, myogenin expression starts in line with a previous data showing that their expression is mutually exclusive (**Figure S5C**) [38]. Control cultures also showed a first expression peak of MEF2D after 72 hours of differentiation as previously described and the expected continuous up-regulation of the more mature differentiation marker nicotinic, cholinergic receptor (**Figure S5D and S5E**) [39]. Scrambled shRNA knock down cultures exhibited the expected expression pattern of muscle markers. In striking contrast, the PKD2-depleted cultures showed substantially reduced expression of all markers mentioned. However, the sequence of the marker expression course was regular (**Figure S5A–E**). Immunoblot analysis also revealed a significantly reduced MHC expression (**Figure 5C and 5D**). MHC was also analyzed by immunocytochemistry. The number of myotubes per visual field was quantified and the fusion indices were calculated (**Figure 5E**). The total number of myotubes per visual field was significantly reduced in PKD2-depleted C2C12 cells (**Figure 5F**). PKD2-4 shRNA-expressing cells showed more single nucleated myocytes indicating that crucial steps required for adequate cell fusion were impaired. In addition, PKD2-depleted cultures exhibited a significantly reduced number of multinucleated myotubes which points to an even more drastic effect during further myotube formation (**Figure 5G**). Thus, PKD2 appears to be required for *in vitro* myoblast differentiation. The residual, but substantially reduced differentiation in the PKD2-4 shRNA-expressing cells could be explained either an induction of other myogenesis-inducing pathways, the presence of residual PKD2 protein or an activation of compensatory mechanisms induced by other PKD isoforms.

### PKD2-depleted cells show normal proliferative capacity

Pharmacological inhibition of PKCs and PKDs using chemical inhibitors revealed to some extent cell death in the differentiating cultures. To dissect whether this is due to reduced PKD activity or can be attributed to inhibitor off-target effects, we examined the effect of PKD2 depletion on cell cycle progression. A cell viability assay showed no difference in the PKD2-depleted cells compared to control cultures (**Figure S6A**). Cell cycle analysis showed also similar profiles in all three lines (**Figure S6B–D**) albeit there was a slight but not significant trend of fewer cells being in S-phase in the #PKD2-4 cell line (**Figure S6B and S6D**). Previously it has been shown that PKDs affect proliferation via interfering with cyclin D levels [40]. However, we could no detect differences in cyclin D1 levels in all our experiments between control and PKD2-depleted cells (**Figure S6E**).

**Figure 6:**
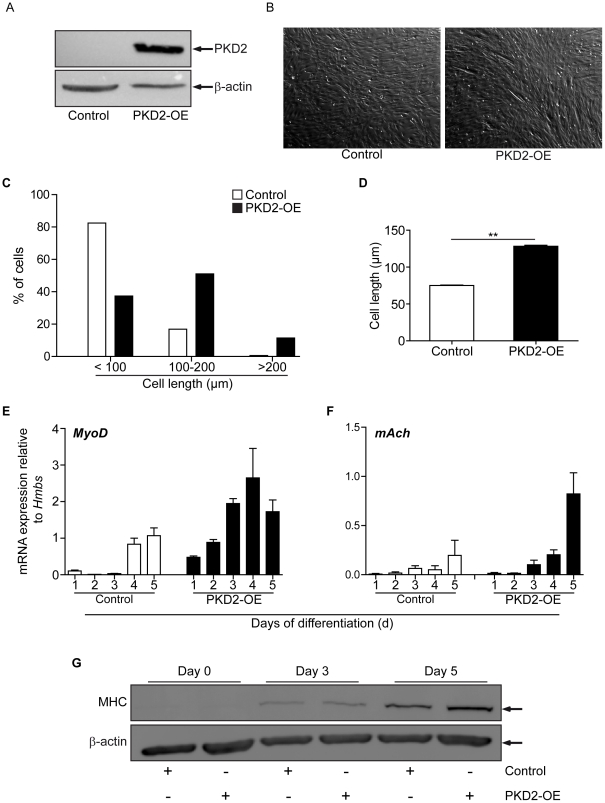
Ectopic expression of PKD2 increased myogenic differentiation. (**A**) C2C12 cells were infected with retroviruses encoding for either EGFP or PKD2 according to *material and methods* After infection cells were selected for one week with neomycin to generate stable and pure cell lines and analyzed via immunoblot for expression of the viral PKD2-transgene. To avoid overexposure and interference with the neighbouring band a short exposure time was chosen in purpose. (**B**) Representative bright field view of control virus transduced C2C12 cells (left photograph) and PKD2 overexpressing C2C12 cells (right photograph) are shown. Scale bars, 20 µM. (**C**) The percentage of cells in the respective group was calculated with the following total numbers of counted cells: control, n = 2421; PKD2-OE, n = 2090 (n = 3). Cells were grouped according to their cell length (<100, 100–200, >200 µM). Numbers of cells per group were as follows: <100: 82% vs. 37%; n = 1996 vs. n = 782. 100–200 µM: 16% vs. 51%; n = 408 vs. n = 1067. >200 µM: 0.6% vs. 11%; n = 16 vs. n = 240. (**D**) Calculated means of all measured cells belonging to either control or PKD2-OE cells are shown. (**E** and **F**) qPCR analysis for MyoD (**E**) and mAch (**F**) expression in stable C2C12 cells during differentiation at indicated time points. (**G**) Control cells and PKD2 transduced cells were differentiated and collected as indicated in the figure. Subsequent immunoblot analysis revealed increased MHC expression in knock down cells. One representative immunoblot is shown.

### PKD1 and PKD3 are less critical for C2C12 myoblast differentiation than PKD2

To exclude potential bias from other PKD isoforms, we generated lentiviral knock down C2C12 cell lines (**Figure S7A–B**). PKD1- and PKD3 depleted cell lines were differentiated as in the previous experiments for PKD2. We chose MyoD as a marker for the initiation of myogensis and mAch as a late marker to assess the mature phases of C2C12 myoblast differentiation. We could not detect a significant difference of MyoD expression in PKD1– and PKD3-depleted cells compared to scrambled infected C2C12 cells on day 3 and 5 (**Figure S7C**) pointing to less important role of these kinases during early muscle differentiation. mAch expression was similar on day 3 while on day 5 PKD3-depleted cells showed significantly less mAch. Given the expression profiles for PKD1, PKD2 and PKD3 (**Figure 1C–E**) these data are perfectly in line with our findings pointing to a less important role of PKD1 throughout C2C12 cell differentiation while PKD3 becomes more critical towards the end of differentiation. However, further experiments have to clarify these issues in more detail.

### Ectopic expression of PKD2 increases myogenic differentiation

To examine whether PKD2 alone could trigger myogenic differentiation of C2C12 cells, we inserted wild type PKD2 into a retroviral vector to induce forced expression of PKD2 (PKD2-OE; **Figure 6A**). Next, we differentiated empty vector- and PKD2-transduced C2C12 cells in our standard myogenic differentiation assay. Forced PKD2 expression accelerated myoblast fusion and increased the number of elongated cells resembling myotubes per visual field. This was already visible on day 2 and continued until the end of the experiment (**Figure 6B**). To assess that in a more rigorous way, we measured cell length in randomly selected cells (Control, n = 2421; PKD2-OE, n = 2090; three independent experiments) without further facilitation via MHC-staining. To define cell length in vector and PKD2-OE cells in greater detail we defined three groups that exhibited <100, 100-200, >200 µM cell length. Most of the control cells could be allocated to the group that contained <100 µM cells while there were less PKD2-OE cells in that group (**Figure 6C**; 82% vs. 37%; n = 1996 vs. n = 782). This is in contrast to the second group where PKD2-OE cells were predominantly found (**Figure 6C**; 16% vs. 51%; n = 408 vs. n = 1067). In the third group (>200 µM) only a few control cells could be found while PKD2-OE cells were the predominat cell type (**Figure 6C**; 0,6% vs. 11%; n = 16 vs. n = 240). Next, we calculated the overall cell length of all three groups and found significant longer cells in the PKD2-OE cells in line with increased differentiation (**Figure 6D**). As a more quantitative approach MyoD and mAch (nicotinic, cholinergic receptor) expression, was determined during differentiation of C2C12 cells via qPCR (**Figure 6E and 6F**). MyoD serves hereby as an early initiation marker and mAch as a mature marker for myotubes[35], [36], [37], [38], [39]. In line with our morphological observations we detected increased and accelerated MyoD (**Figure 6E**) expression as well as more mAch (**Figure 6F**) in particular at later time points in C2C12 cells over-expressing PKD2. Immunoblot analysis revaled a slightly increased level of myosin heavy chain on day 3 with a marked increase on day 5 (**Figure 6G**). These data indicate that expression of PKD2 is sufficient to accelerate myogenic differentiation.

### PKD2 contributes to myogenic differentiation of primary satellite cells

**Figure 7:**
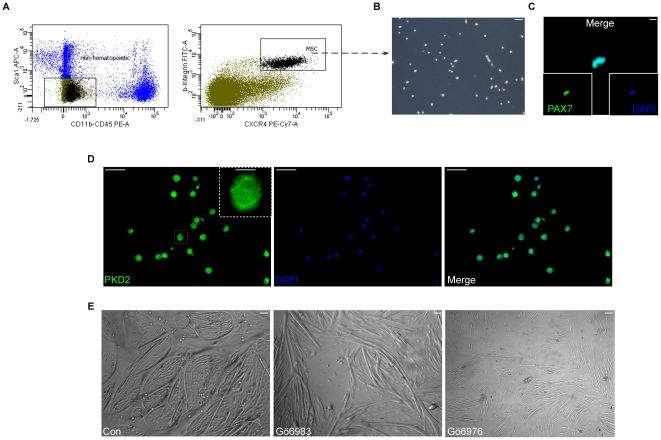
Pharmacological inhibition of PKD activity reduces myogenic differentiation of primary satellite cells. (**A**) Schematic overview of the performed FACS-sorting regimen. Sca1, CD11b and CD45 positive cells were excluded as hematopoietic cells. Cells being negative for this marker population were gated as shown in the left panel. Gated cell were further analyzed for positive staining of CXCR4 and β1-integrin. Double positive cells were considered as satellite cells and FACS-sorted. (**B**) FACS-sorted cells after one day of culture in vitro. Scale bars, 100 µM. (**C**) Satellite cells stain positive for nuclear Pax7 (green). Nuclei are visualized by DAPI and are stained in blue color. Scale bar, 12.5 µM. (**D**) Satellite cells express PKD2. PKD2 staining is shown in green, nuclei in blue. Scale bars, 37.5 µm. Insert shows higher magnification of single cell marked by the dashed square. Arrows point on perinuclear localization of PKD2. Scale bar, 12.5 µM. (**E**) Cells were seeded on laminin coated cover slips and treated with either Gö6983 or Gö6976 (both 10 µM). Bright field images were done after 2 days of initiation of differentiation. PKD inhibition in primary satellite cells showed similar morphology with disrupted myotube fusion as C2C12 cells Scale bar, 100 µM.

To corroborate the data obtained in C2C12 cells, we subsequently investigated the role of PKDs in primary isolated murine satellite cells. A unique combination of cell-surface markers (*C*D45^−^ *S*ca-1^−^ *M*ac-1^−^CXCR*4* ^+^ *β*1-integrin^+^) defines autonomously myogenic cells within the myofiber-associated satellite cell compartment of adult mouse skeletal muscle and allows their direct isolation by fluorescence-activated cell sorting (FACS) [41]. First, we isolated this particular cell type according to its surface marker profile by FACS-sorting (**Figure 7A**). Isolated cells exhibited the typical morphology of primary satellite cells cultured *in vitro* (**Figure 7B**) and robust expression of nuclear Pax7 protein (**Figure 7C**), a selective marker of primitive myogenic cells. PKD2 expression in primary satellite cells was verified by immunostaining (**Figure 7D**). After initiation of differentiation by FGF- and serum withdrawal, the cells were subsequently incubated with either Gö6976 or Gö6983. We observed similar morphological changes as in C2C12 cells including disrupted myotube fusion upon treatment with the inhibitor. This observation points to a pivotal role of the PKC/PKD pathway in myogenesis (**Figure 7E**).

### PKD2 induces activation of Mef2D

**Figure 8:**
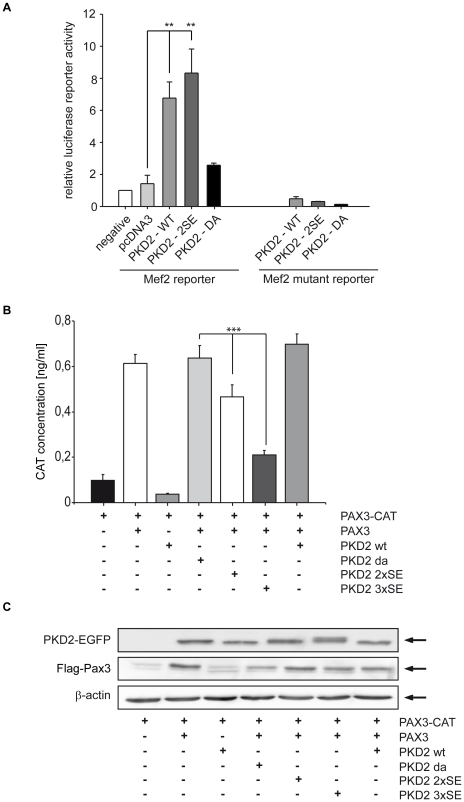
PKD2-induced myogenesis is mediated via Mef2D activation and Pax3 repression. (**A**) C2C12 cells were co-transfected with Mef2D-luc or Mef2D-luc mutated, Renilla and either wild type (PKD2-WT), catalytically active mutant (PKD2-S^706/710^E) or catalytically inactive (PKD2-DA) PKD2 constructs, respectively. Medium change from growth to differentiation medium occurred after 36 hours. After incubation with differentiation medium for 24 hours, cells were harvested and luciferase assays were performed according to the manufacturer's instructions. Graphs were generated out of three independent and tested for significance using the Student *t*-test. P<0.05 (*) and p<0.01 (^**^) are indicated. (**B**) Cells were co-transfected with pcDNA3-Pax3-Flag, (PRS-9)-TK-CAT and/or pEGFP-PKD2 mutants as indicated. 24 hours upon transfection cells were harvested and CAT ELISA was performed according to the manufacturer's instructions. Graphs were generated out of three independent experiments and tested for significance using the Student *t*-test. P<0.001 (***).

Previously we have shown that PKD2 can activate Mef2D in gastric cancer cells via phosphorylation of class II HDACs [14]. Mef2D is a transcription activator that binds specifically to the MEF2 element present in the regulatory regions of many muscle-specific genes and controls cardiac morphogenesis and myogenesis. Therefore, we hypothesized that Mef2 transcriptional regulators could be potential myogenic targets of PKD2 in muscle precursor cells. Exogenous expression of wild-type PKD2 or active PKD2 in C2C12 myoblasts resulted in a 4.5- and 7-fold induction of Mef2 promotor activity in C2C12 cells, respectively (**Figure 8A** **)**. The already high level of Mef2 promotor activity in cells expressing PKD2 wild type is likely to be due to the fact that exogenous expression of wild-type PKD2 in C2C12 cells results in considerable activation of the kinase (data not shown). In contrast, there was no significant increase in Mef2 promoter activity compared to the expression of the empty vector plasmid when cells expressed catalytically inactive PKD2 or a Mef2D reporter plasmid containing mutated ME2D binding sites.

### PKD2 inhibits transcriptional activity of Pax3

*Pax* genes have been shown to promote self-renewal and population expansion in undifferentiated myoblasts. In C2C12 cells it has been shown that Pax3 overexpression increases proliferation capacity and delays myogenic differentiation [42], [43], [44]. We have demonstrated that PKD2 activates the pro-myogenic transcription factor Mef2D. Next we examined whether PKD2 was able to inhibit Pax3 transcriptional activity as another mechanism to promote myogenic differentiation. To address this question we performed Pax3 reporter assays in C2C12 cells expressing different PKD2 mutants and Pax3. Exogenous expression of wild-type PKD2 or catalytically inactive PKD2 (PKD2-DA) did not decrease Pax3 reporter activity. However, coexpression of constitutively active PKD2 mutants [14], [15] in C2C12 myoblasts resulted in a reduction of 30% and 70% of Pax3 transcriptional activity, respectively, compared to PKD2 wild-type and to a catalytically inactive mutant (**Figure 8B and 8C**). Thus PKD2 can regulate myogenic differentiation by affecting two transcriptional mechanisms, activation of Mef2D and inhibition of Pax3 transcriptional activity.

## Discussion

Muscle differentiation is regulated by an orchestrated network of transcription factors that basically constitute two families; the MyoD group bHLH muscle regulatory factors (MRFs) and the MEF2 group of MADS-box regulators. It is a tightly controlled process and can be induced *in vitro* by serum deprivation, activation of RTKs, such as the insulin- or the IGF-1 receptor, or by ligands of heptahelical receptors, such as S1P. At the signaling level, various signaling pathways, including PLCβ1 and PLCγ1, have been implicated as mediators of insulin-induced C2C12 myoblast differentiation [45], [46], [47]. Recently it has been demonstrated that PKCε is up-regulated and its activity increases during muscle differentiation. Furthermore, PKCε and PLCγ1 were shown to form a complex and were found to be crucial for insulin-induced myogenic differentiation. In addition, both proteins co-localized with the Golgi marker 58-K in C2C12 cells [45]. PLC enzymatic activity results in the production of inositol 1,4,5-trisphosphate and diacylglycerol (DAG). DAG activates not only DAG-sensitive PKC isoforms including PKCε, but also directly PKDs including PKD2 [48], [49]. In addition, PKDs are prominent downstream targets of PKCs, in particular novel PKCη and ε. On the other hand, the highly expressed class II HDACs in skeletal muscle were known to repress the expression of MEF2-dependent genes by directly binding to MEF2. The loss of HDAC-MEF2 binding followed by the export of HDACs from the nucleus to the cytoplasm is mediated by the members of CaMK family. However, later studies have shown that PKDs were the crucial players in this process by serving the role of downstream effector kinases of PKCs and by stimulating the nuclear export of class II HDACs [50]. Considering the critical role of HDACs in regulating myocyte differentiation and remodeling and PKCs in the signal transduction pathways of muscle differentiation, PKDs could play an important role in muscle differentiation. Our data show that C2C12 myoblasts express all PKD isoforms with PKD2 being the predominant isoform expressed during the entire course of differentiation: PKD2 was phosphorylated/activated during the initiation of C2C12 myoblast differentiation. Selective inhibition of PKCs or PKDs as well as depletion of PKD2 by specific shRNA constructs resulted in a marked inhibition of the expression of muscle differentiation markers, such as MyoD, Myogenin, Mef2D, Pax7 and MHC and myotube formation, respectively. This statement was further supported by data wherein the differentiation of primary satellite cells was inhibited by treatment of either PKC or PKD inhibitor. Thus, PKD2 appears to be required for differentiation of C2C12 myoblasts.

The obtained expressions with our two independent PKD2 knock down cells are from particular interest. In fact it is noteworthy that the alteration in myogenic genes during differentiation was dose-dependent in terms of PKD2 expression. The knock down in cell line #PKD2-2 was less efficient than in cell line #PKD2-4 while the loss of the myogenic markers was greater in cell line #PKD2-4. However, our expression data clearly show that the myogenic program was not entirely disrupted since e.g. myogenin and nicotinic cholinergic receptor were still up-regulated. However, this is not surprising. The myogenic program is highly conserved and tightly regulated [6]. The four key factors-MyoD, myogenin, Myf5 and MRF4 act at multiple points in the skeletal muscle lineage to establish the skeletal muscle phenotype [6] and thereby serve as the most crucial players during skeletal muscle myogenesis. However, both knock out mice of MyoD and Myf5 lack any myogenic phenotype [6], [51]. Given the fact that even MyoD and Myf5 are functionally redundant it is not surprising that myoblast differentiation is not finally governed by just one kinase.

On the other hand, we observed a differential expression pattern for the other two PKD isoforms. While PKD1 was expressed at very low levels during the entire course of differentiation, PKD3 was significantly upregulated at the terminal stage of differentiation. In accordance, knockdown of PKD1 and PKD3 did not display any significant effect on differentiation as judged by the expression of marker gene-MyoD with the exception being the effect of PKD3 knockdown on the expression of a late differentiation maker gene, mAch. However, this is explained by the fact that PKD3 is up regulated at the terminal stages of differentiation and therefore could play a role at this stage.

PKDs are major regulators of vesicle fission at the *trans*-Golgi network (TGN) in many cells [52] and are targeted to the Golgi by class I ARF proteins [53] where they are activated by local DAG and βγ subunits [54]. In C2C12 cells, PKD2 could be activated via two ways, indirectly via PKCε [55], which is also active during myogenic differentiation [45] or directly via PLC-mediated production of DAG. We identified S1P as a potential physiological activator of PKD2 in C2C12 cell myogenic differentiation.

Nevertheless, it is noteworthy that PKD2 depletion strongly alters myogenic differentiation as shown at the level of morphology, gene expression and finally on protein level. Vice versa, we found increased myogenic differentiation upon overexpression of PKD2 in C2C12 myoblasts. PKD2 activates Mef2Ds. It has been shown previously that MEF2 factors and the myogenic bHLH factors such as MyoD each regulate the expression of a number of contractile protein genes. Thereby interaction between Mef2 proteins and the myogenic bHLH proteins is important in directing muscle gene expression [2], [3], [4], [6], [51], [56], [57]. Furthermore, we show, for the first time, that PKD2 negatively regulates Pax3 transcriptional activity. Pax3 has been shown to prevent differentiation and increase self-renewal of C2C12 myoblasts in a variety of studies [42], [43], [44], [58]. Thereby, we propose that PKD2 functions in a dual mode of myoblast differentiation (i) repression of self-renewal and (ii) induction of differentiation. Taken together, these data identify PKDs, in particular PKD2 as a novel regulator of muscle differentiation downstream of PKCε and PLCγ and the S1P receptor and thereby, as a potential novel target for the modulation of muscle regeneration in myoblasts.

## Materials and Methods

### Cell lines and reagents

C2C12 cells were obtained from the European Collection of Cell Cultures (Salisbury, UK) and cultured in DMEM (Invitrogen, Karlsruhe, Germany) supplemented with 20% FCS (PAA, Cölbe, Germany) and 1% Penicillin/Streptomycin (Invitrogen). Cells were induced to differentiate at 80–90% confluence by transferring them into DMEM plus 2% horse serum (differentiation medium, [DM]), 1% Penicillin/Streptomycin and 1% Glutamax (Invitrogen). The inhibitors Gö6976, Gö6983 as well as the phorbol ester PMA were purchased from Calbiochem, Bad Soden, Germany. Treatment of the inhibitors started with the switch from growth to differentiation medium. Medium was replaced every other day.

### Western blot analysis

Immunoblot analysis was performed according to standard protocols. C2C12 cells were harvested and dissolved either in lysis buffer A (100 mM Tris-HCl, pH 6.8, 2% SDS, 10% glycerol, 0.01% phenol red, 100 mM dithiothreitol, protease inhibitor tablet, Roche) or lysis buffer B (50 mM Tris/HCl, pH 7.6, 2 mM EGTA, 2 mM EDTA, 1% Triton x-100, 2 mM DTT, protease and phosphatase inhibitor cocktail. Whole cell extracts (50 µg to 100 µg) were subjected to gel electrophoresis (SDS-PAGE) and transferred to PVDF membranes (Millipore Corporation, Bedford, USA). Membranes were blocked using 5% dry milk or BSA (for phospho-PKD antibody) in phosphate-buffered saline containing 0.1% Tween 20. For subsequent washes, 0.1% Tween 20 in phosphate-buffered saline was used. Membranes were incubated o/n with specific antibodies at 4°C under shaking conditions. To detect phosphorylated PKD we used a pPKD Ser744/748 antibody (1∶1000; Cell Signaling, Danvers, USA). Anti-PKD1 antibody (1∶1000) was purchased from Santa Cruz Biotech. Inc., Santa Cruz, USA). Anti-PKD2 and 3 antibodies (1∶1000) were from Bethyl Laboratories Inc., Montgomery, USA). Myogenin and MHC antibodies were purchased from the Developemental Hybridoma Bank (1∶1000; University of Iowa, Iowa City, USA). MyoD antibody (1∶500) was obtained from Santa Cruz Inc. Chemiluminescence was detected using a Chemi Smart Chemiluminescence 5000 documentation system (PeqLab Biotechnologie GmbH, Erlangen, Germany).

### Densitometric analysis

Densitometric analysis was performed by using BioProfil BIO-1D software, version 12.04 (Vilber Lourmat Deutschland GmbH, Eberhardzell, Germany). Relative band intensity of each time point was measured and set in relation to either PKD2 or actin loading control as indicated in the figure legend.

### Immunoprecipitation

Immunoprecipitations were performed as described previously[53]. Briefly, C2C12 cells were lysed in lysis buffer A. After centrifugation at 14,000 rpm for 10 min, protein concentrations were measured in the lysates. 2000 µg of extracts were incubated with 2 µg of either control IgG antibody (Santa Cruz Inc) or PKD2 antibody at 4°C and after 1 h, 80 µl of protein A sepharose beads were added and incubated for 1 h. Immobilized proteins were washed extensively, and resuspended in Laemmli buffer and subjected to SDS–PAGE and western blotting, as described above.

### Quantitative one-step real-time RT-PCR (qPCR)

One-step real-time qPCR was carried out with the LightCycler® System (Roche, Mannheim, Germany) using the QuantiTect SYBR Green RT-PCR kit (Qiagen, Hilden, Germany) and was done as previously described [59]. Briefly, to verify the specificity of the PCR amplification products, LightCycler melting curve analysis was performed. Relative transcript expression was expressed as the ratio of target gene concentration to the housekeeping gene hydroxymethylbilane synthase (*Hmbs*) concentration. QuantiTect primer assays (Qiagen) were used in all experiments.

### Immunocytochemistry

Immunofluorescence analysis was carried out with cells cultivated on coverslips. The cells were rinsed in PBS and fixed for 10 min with PBS containing 4% paraformaldehyde at room temperature. For MHC/F-actin/DAPI co-staining, cells were incubated for 10 min in 50 mM NH_4_Cl and subsequently treated for 10 min with 0.1% Triton X (Sigma-Aldrich, Steinheim, Germany) followed by 30 min incubation in 0.1% Goat serum plus 1% fish skin gelatin (Sigma-Aldrich) for blocking. Then, preparations were incubated with the primary antibody against MHC (1∶200; mouse anti-MF20 IgG, Developmental Hybridoma Bank) in a humidified chamber o/n at 4°C. After washing three times in PBS, cells were incubated for one hour at 37°C with an Alexa 488-labeled rabbit anti-mouse IgG antibody and Alexa 568-conjugated phalloidin (1∶50; Invitrogen). Afterwards, cells were washed in PBS again and treated with DAPI 1∶5000 for 5 min. Samples were subsequently washed and embedded in Vectashield mounting medium (Vector, USA) and analysed by fluorescence microscopy. For Pax7 (1∶50; Developmental Studies Hybridoma Bank) staining of primary isolated satellite cells, cells were spotted on a glass slide. After drying the cells at room temperature, cells were fixed for 10 min with PBS containing 4% paraformaldehyde. Mouse antigens were blocked using the MOM kit (Vector Labs) and permeabilized using Triton-X. Pax7 antibody was incubated overnight at 4°C. After washing the preparations three times in PBS, cells were incubated for one hour at 37°C with an Alexa 488-labeled rabbit anti-mouse IgG antibody. Imaging was performed with a Keyence inverted fluorescence microscope with filters detecting excitations at 360 (Hoechst), 490 (Alexa Fluor 488), and 560 (Alexa Fluor 568) and a 60x oil objective. Images were processed using ImageJ (http://rsbweb.nih.gov/ij/).

### Lentiviral shRNA delivery and generation of stable cell lines

All short hairpin shRNAs used in this study were constructed in pLKO.1-puro. shRNAs for PKD2 were purchased from Sigma-Aldrich (MISSION® shRNA). Each gene set contained four constructs with distinct target sequences, all of which were packaged for viral production and infection, and tested for target knockdown. For viral packaging, pLKO-shRNA, pCMV-dR8.91 and pCMV-VSV-G were co-transfected into 293T cells using Fugene 6 (Roche) at 3 µg, 2,7 µg and 0,3 µg respectively (in 7 ml for a 10 cm dish). Two rounds of transduction were carried out with supernatant collected either after 24 or 48 hours in the presence of 8 µg/ml polybrene (Sigma-Aldrich). Subsequently, the cells were selected for 5 days in growth medium containing 3 µg/ml puromycin [9]. For depletion of PKD1 and 3, hairpins were cut from the pSM2 Retroviral shRNAmir libraries (Open Biosystems, Huntsville, USA) and cloned into the pGipz vector (Open Biosystems). Three hairpins targeting either PKD1 or PKD3 were then pooled and lentiviral particles were generated as described above. Infected cells were purified with puromycin (3 µg/ml). A pGipz vector harboring a scrambled shRNA served as a control.

### Retrovial infection and generation of stable cell lines

For retroviral packing pCL-Eco plasmid was co-transfected together with PKD2-MSCVneo plasmid in 293T cells. Two rounds of transduction were carried out with supernatant collected either after 24 or 48 hours in the presence of 8 µg/ml polybrene (Sigma-Aldrich). Subsequently, the cells were selected for 5 days in growth medium containing 800 µg/ml neomycin.

### Transient transfections and reporter assays

C2C12 cells were transfected with Lipofectamine (Invitrogen) according to the manufacturer's instructions. Luciferase activity was determined using the Dual Luciferase Assay Kit (Promega, Mannheim, Germany). Firefly luciferase relative light units were normalized with renilla luciferase activity after co-transfection of a pRL-TK plasmid (Promega). MEF2D reporter assays were performed by co-transfecting 100 ng MEF2D and 2 µg of the following vectors: PKD2-wild type, PKD2-S^706/710^E double mutant and catalytically inactive PKD2 mutant (PKD2-DA) [60]. The expression level of reporter-only transfected C2C12 cells was used as reference luciferase activity and set to 1.

### Myotube quantifications

Cells were stained for Myosin heavy chain (MHC) and DAPI and 10 randomized visual fields per condition were photographed in three independent experiments. Each MHC positive cell per visual field was counted and defined as myotube. Fusion index was built by counting manually nuclei of the MHC positive cells and dividing them into three different groups: Myotubes containing but one nucleus, two to three nuclei and more than three nuclei.

### Measurement of cell length

To determine cell length in differentiating PKD2-OE versus control C2C12 cells, 30 randomly selected high magnification images were taken from three independent experiments. The percentage of cells in the respective group was calculated with the following total numbers of counted cells: control, n = 2421; PKD2-OE, n = 2090 (n = 3). Cells were grouped according to their cell length (<100, 100-200, >200 µM).

### Cat ELISA

To examine the effect of PKD2 on Pax3 transcriptional activity C2C12 cells were transfected with the Lipofectamine LTX (Invitrogen) according to the manufacturer's specifications. The LTX precipitate consisted of a combination of 1 µg of pcDNA3-Pax3-Flag, 2 µg of the (PRS-9)-TK-CAT reporter plasmid, containing the Pax3 paired and homeodomain recognition sequences, and 5 µg of pEGP-PKD2 mutants. Analysis of chloramphenicol acetyl transferase (CAT) level was performed using the CAT ELISA Kit (Roche) according to the manufacturer's specifications. All samples were corrected for the transfection efficiency with respect to protein concentration. For protein determination, Lowry and Bradford assays were used.

### Satellite cell isolation

Satellite cells were prepared from hindlimb muscles (tibialis anterior muscle, tibialis gastrocnemius muscle, soleus muscle) of 2–3 month old mice. Briefly, muscles were digested in DMEM (Invitrogen)/0.2% Collagenase Type II (Invitrogen) for 90 minutes, the muscle tissue was then mechanically dissociated with a Pasteur pipette. After several steps of sedimentation and washing, the myofibers were again enzymatically treated with 0.0125% Collagenase and 0.05% Dispase (Invitrogen) for 30 minutes to release satellite cells. Subsequently, satellite cells were centrifuged, filtered (70 micron) and stained with the following markers: CD11b^-^ PE, CD45^-^ PE (1∶200; eBioscience, San Diego, USA), Sca-1^-^ APC (1∶800; eBioscience), CXCR4^+^ bio (1∶100; BD Pharmingen, Heidelberg, Germany) and β1-integrin^+^ (1∶200; BD Pharmingen) purified, Streptavidin-PE-Cy7 (1∶100; eBioscience), anti-mouse IgG-FITC (1∶100; eBioscience) [41]. Living cells were labelled by positive staining for calcein blue (1∶1000, Invitrogen) and negative staining for propidium iodide (PI, 1 µg/mL). FACS sorting was performed at a FACS Aria II cell sorter (Becton Dickinson).

### Satellite cell culture and differentiation

Prior to plating (24 hr), plates were coated with 0.2% rat-tail collagen and 5 µg/mL natural mouse laminin (Invitrogen). Cells were plated at 1×10^4^ cells/cm^2^ in growth medium in either 24-well tissue culture plates. Growth medium was composed of Ham's F10+20% FBS +5 ng/mL bFGF (Invitrogen) +1% penicillin/streptomycin +1% Gluta-Max. bFGF was replaced daily. After 5–7 days, cells were passaged by aspiration of medium, washing with PBS, and incubation with PBS for 5 min at 37°C. Cells then were replated onto collagen/laminin-coated chamber slides in growth medium for 2 days, and then medium was changed to differentiation medium: Opti-MEM (Invitrogen) +1% FBS +1% penicillin/streptomycin +1% GlutaMax. Inhibitor treatment (Gö6983, Gö6976) was done as described for C2C12 cells.

### Ethical Treatment of Animals

The animal work on primary mouse satellite cells was approved by the government of the state of Baden-Württemberg (Regierungspräsidium Tübingen 35/9185.81-3; Permit number No.919). All efforts were made to minimize suffering of the animals. No experiments were performed on living animals.

### Statistical analysis

For all figures in this study, if not stated otherwise, standard errors of the mean (s.e.m.) are indicated by error bars, and *p-*values by student t-test <0.05, 0.01 or 0.001 are indicated by either one, two or three asterisks, respectively. In case of using Wilcoxon statistical test, p-values <0.05 or 0.01 are indicated in the figure legend, respectively.

## Supporting Information

Figure S1 C2C12 cell differentiation recapitulates myogenesis in vitro. Wild type C2C12 cells were seeded on day -1 to achieve a confluency of approximately 70% at day 0. Differentiation of C2C12 cells was induced by change of medium from growth medium to differentiation medium. At day 1 of differentiation, cells become more confluent and start developing alignment. On day 3 of differentiation, first small myotubes are detectable. At day 6 of differentiation, myotubes become more elongated, multinucleated and ramified. Scale bar, 100 µM. Shown is one representative serie of bright field photographs out of 6 independent identical experiments.
(11.56 MB TIF)

Figure S2 Specificity of PKD2 antibody detecting only PKD2 but not PKD1 or PKD3. Hek-293T cells were transfected with expression vectors harboring GFPfusion proteins with each PKD isoform. The respective lysates were subsequently analyzed via immunoblotting with PKD2 antibody. Upper band shows exclusive detection of the EGFP-PKD2 fusion protein in cells transfected with the respective plasmid. No signals were detected in PKD1-GFP and PKD3-GFP transfected variants. All groups showed endogenous PKD2 expression as shown by the weak band (low intensity band). (*) marks unspecific bands.
(1.38 MB TIF)

Figure S3 Pharmacological inhibition of PKCs and PKDs inhibits myoblast differentiation in vitro. (A) Cells were seeded on cover slips and treated with either Gö6976 or Gö6983. Concentrations are indicated in the figure. DMSO served as a solvent control and did not affect myogenic differentiation of C2C12 cells. On day 5, cover slips were stained for MHC (green), f-actin (red) and nuclei (blue). Photographs are representative for 3 independent experiments. Scale bars, 20 µM. (B) Number of myotubes or MHC positive cells per visual field. Ten randomly selected visual fields were photographed and number of MHC positive cells were plotted as indicated in the figure. P<0.05 (*), p<0.01 (**), and p<0.001 (***). (C) Fusion indices for inhibitor (Gö6076 and Gö6983)-treated cultures in comparison to solvent controls are shown. Concentrations are indicated in the figure. Fusion indices were calculated as the number of nuclei per myotube and sub-classified as 1 nucleus per myotube (upper left panel), 2-3 nuclei per myotube (upper right panel) or more than 3 nuclei per myotube (lower panel). Ten randomly selected visual fields were evaluated. P<0.05 (*), p<0.01 (**), and p<0.001 (***).
(5.97 MB TIF)

Figure S4 PKC and PKD inhibitors alter myotube morphology differently. (A-B) Length and width of myotubes per visual fields were measured using Image J. Length-to-width ratio was calculated and mean values were plotted as indicated in the figure for either day 3 (A) or day 5 (B). Ten randomly selected visual fields were evaluated. P<0.05 (*), p<0.01 (**) and p<0.001 (***).
(0.65 MB TIF)

Figure S5 PKD2-depleted cells show altered expression of myogenic markers. Scramble-, shRNA-PKD2 construct #2 and shRNA-PKD2 construct #4 infected cells were differentiated for 5 days and mRNA samples were collected. qPCR for Pax7 (A), MyoD (B), Myogenin (C), Mef2D (D) and mAch [nicotinic, cholinergic receptor, (E)] expression (n = 3).
(1.34 MB TIF)

Figure S6 PKD2-depleted cells show normal proliferative capacity. (A) The CellTiter-Glo Luminescent Cell Viability Assay was used to determine the number of viable cells in cultures based on quantitfication of the ATP present. Values represent measurement of relative light units (RLU) 48 hours after seeding equal numbers of cells (n = 3). (B) Quantification of percentage of cells being in either G1-, S or G2/M-phase (n = 3). (C and D) Representative cell cycle plot for control vs. shRNA PKD2-2 (C) and shRNA PKD2-4 (D). (E) Immunoblot analysis for Cyclin D1 in Control- and PKD2-depleted cells after 24 hours of culture in growth medium. Representative immunoblot out of three independent experiments.
(1.89 MB TIF)

Figure S7 PKD1 and PKD3 are less critical for C2C12 myoblast differentiation. (A-B) Generation of stable C2C12 cell lines after lentiviral infection with either scramble shRNA (Con) or a pool of shRNAs targeting either PKD1 (A) or PKD3 (B). (C) qPCR analysis of MyoD on day 3 and day 5 of differentiation of the indicated cell lines. (D) qPCR analysis of mAch on day 3 and day 5 of differentiation of the indicated cell lines. N = 3; n.s. = not significant.
(1.66 MB TIF)
