# Supplementary figures and images for: Protein Kinase D2 Is an Essential Regulator of Murine Myoblast Differentiation
Source: PLoS One. 2011 Jan 27;6(1):e14599. doi: 10.1371/journal.pone.0014599 (PMC3029294; doi:10.1371/journal.pone.0014599)

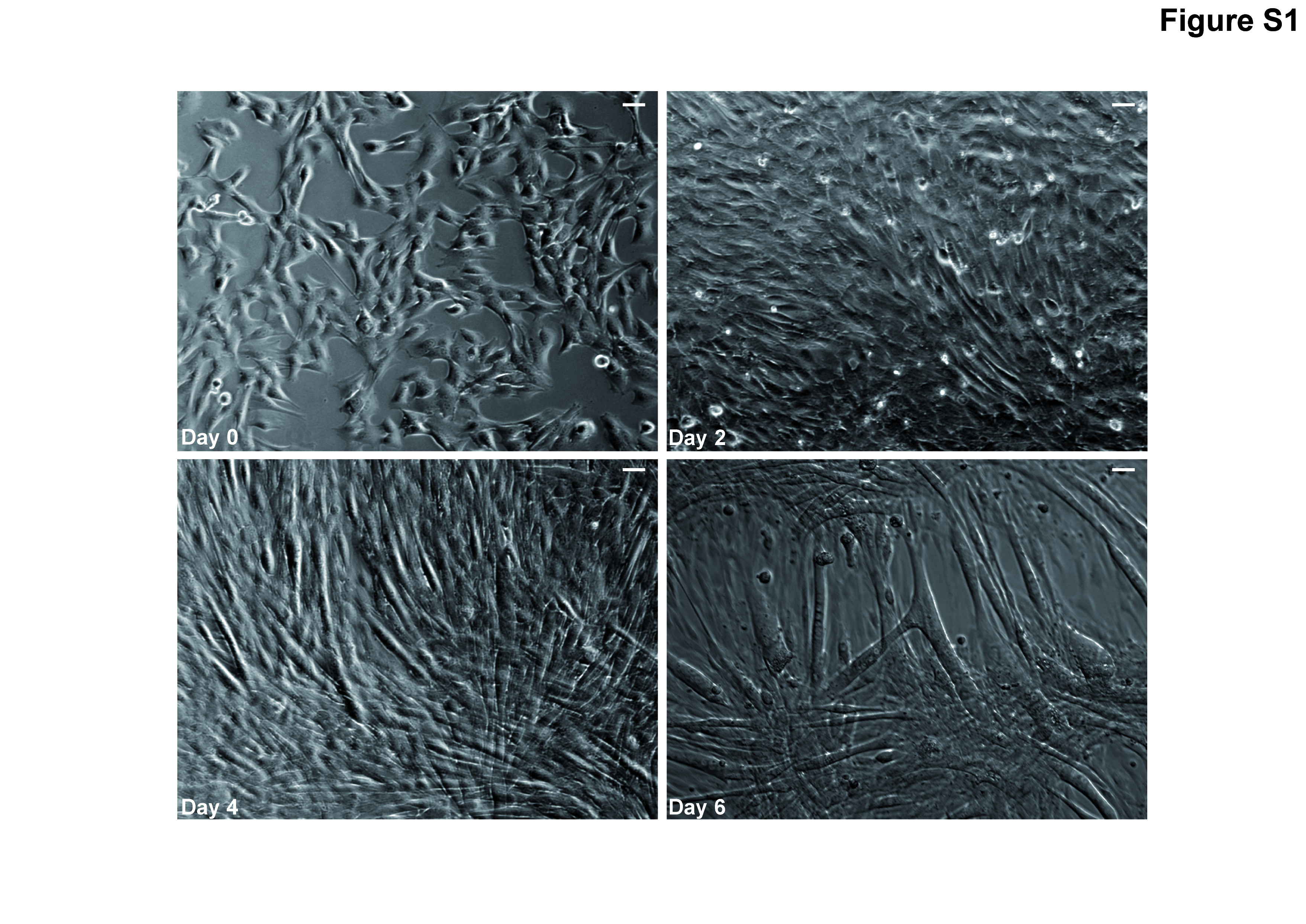

Supplement: Figure S1 — C2C12 cell differentiation recapitulates myogenesis in vitro. Wild type C2C12 cells were seeded on day -1 to achieve a confluency of approximately 70% at day 0. Differentiation of C2C12 cells was induced by change of medium from growth medium to differentiation medium. At day 1 of differentiation, cells become more confluent and start developing alignment. On day 3 of differentiation, first small myotubes are detectable. At day 6 of differentiation, myotubes become more elongated, multinucleated and ramified. Scale bar, 100 µM. Shown is one representative serie of bright field photographs out of 6 independent identical experiments. (11.56 MB TIF) [file pone.0014599.s001.tif]

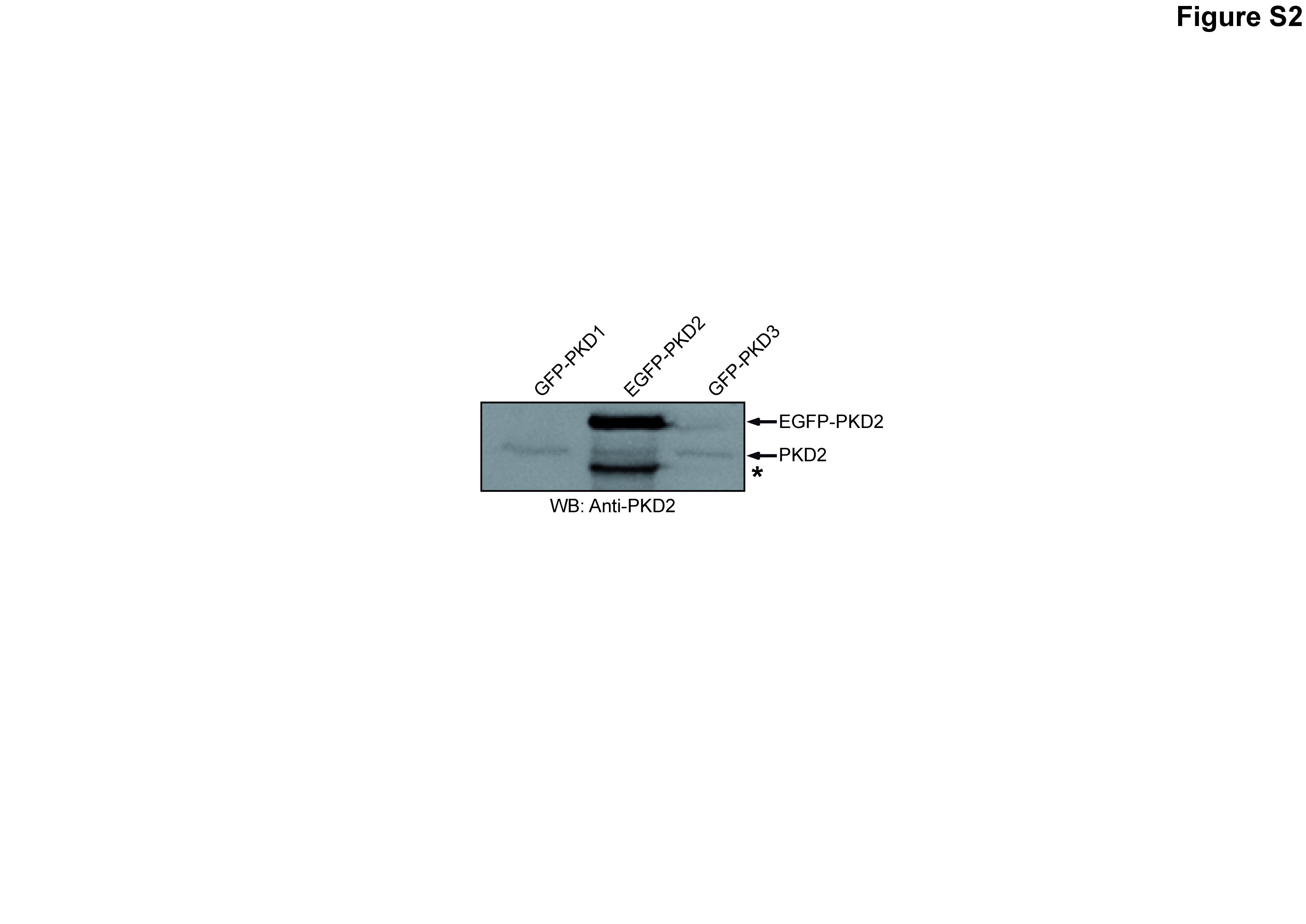

Supplement: Figure S2 — Specificity of PKD2 antibody detecting only PKD2 but not PKD1 or PKD3. Hek-293T cells were transfected with expression vectors harboring GFPfusion proteins with each PKD isoform. The respective lysates were subsequently analyzed via immunoblotting with PKD2 antibody. Upper band shows exclusive detection of the EGFP-PKD2 fusion protein in cells transfected with the respective plasmid. No signals were detected in PKD1-GFP and PKD3-GFP transfected variants. All groups showed endogenous PKD2 expression as shown by the weak band (low intensity band). (*) marks unspecific bands. (1.38 MB TIF) [file pone.0014599.s002.tif]

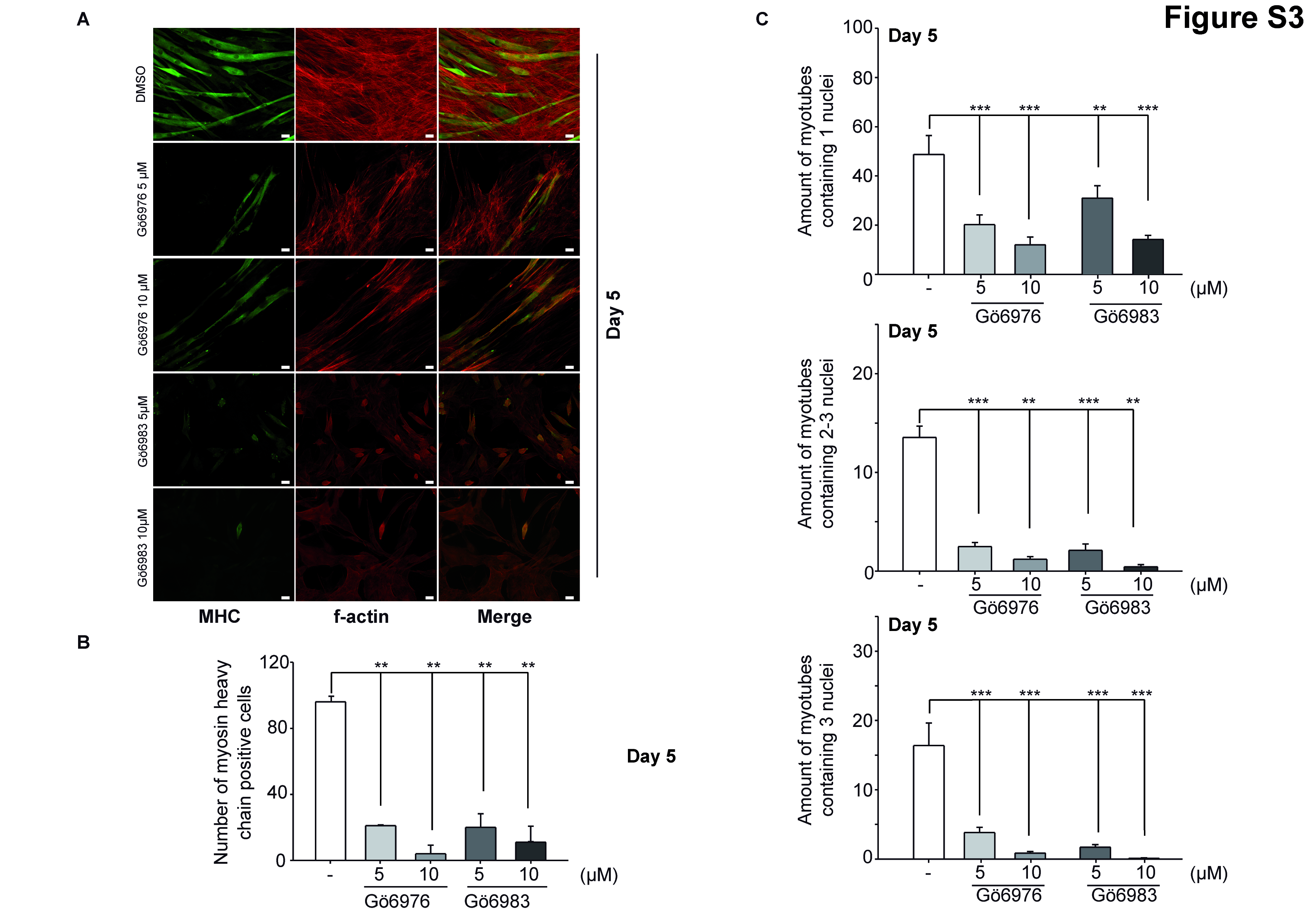

Supplement: Figure S3 — Pharmacological inhibition of PKCs and PKDs inhibits myoblast differentiation in vitro. (A) Cells were seeded on cover slips and treated with either Gö6976 or Gö6983. Concentrations are indicated in the figure. DMSO served as a solvent control and did not affect myogenic differentiation of C2C12 cells. On day 5, cover slips were stained for MHC (green), f-actin (red) and nuclei (blue). Photographs are representative for 3 independent experiments. Scale bars, 20 µM. (B) Number of myotubes or MHC positive cells per visual field. Ten randomly selected visual fields were photographed and number of MHC positive cells were plotted as indicated in the figure. P<0.05 (*), p<0.01 (**), and p<0.001 (***). (C) Fusion indices for inhibitor (Gö6076 and Gö6983)-treated cultures in comparison to solvent controls are shown. Concentrations are indicated in the figure. Fusion indices were calculated as the number of nuclei per myotube and sub-classified as 1 nucleus per myotube (upper left panel), 2-3 nuclei per myotube (upper right panel) or more than 3 nuclei per myotube (lower panel). Ten randomly selected visual fields were evaluated. P<0.05 (*), p<0.01 (**), and p<0.001 (***). (5.97 MB TIF) [file pone.0014599.s003.tif]

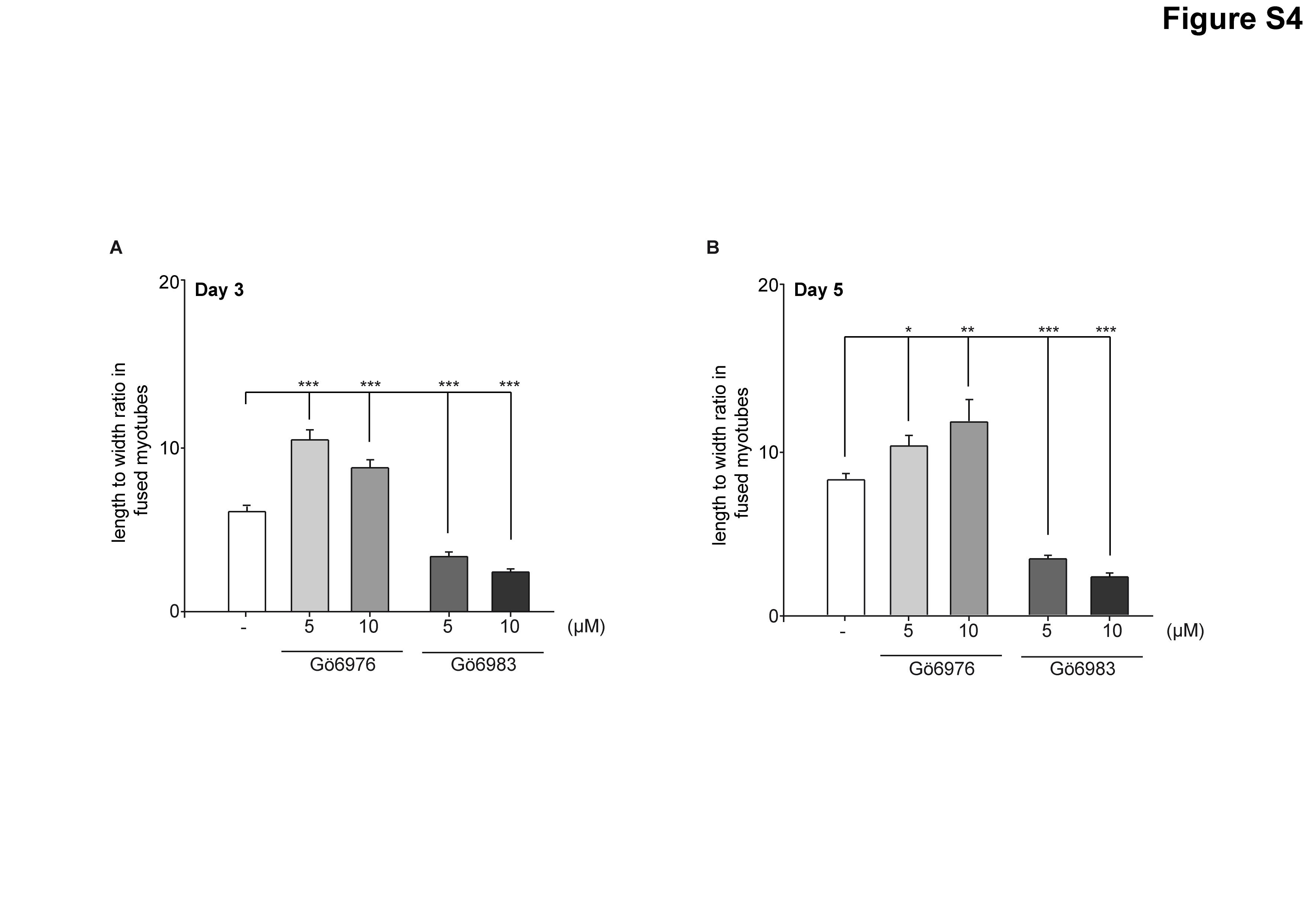

Supplement: Figure S4 — PKC and PKD inhibitors alter myotube morphology differently. (A-B) Length and width of myotubes per visual fields were measured using Image J. Length-to-width ratio was calculated and mean values were plotted as indicated in the figure for either day 3 (A) or day 5 (B). Ten randomly selected visual fields were evaluated. P<0.05 (*), p<0.01 (**) and p<0.001 (***). (0.65 MB TIF) [file pone.0014599.s004.tif]

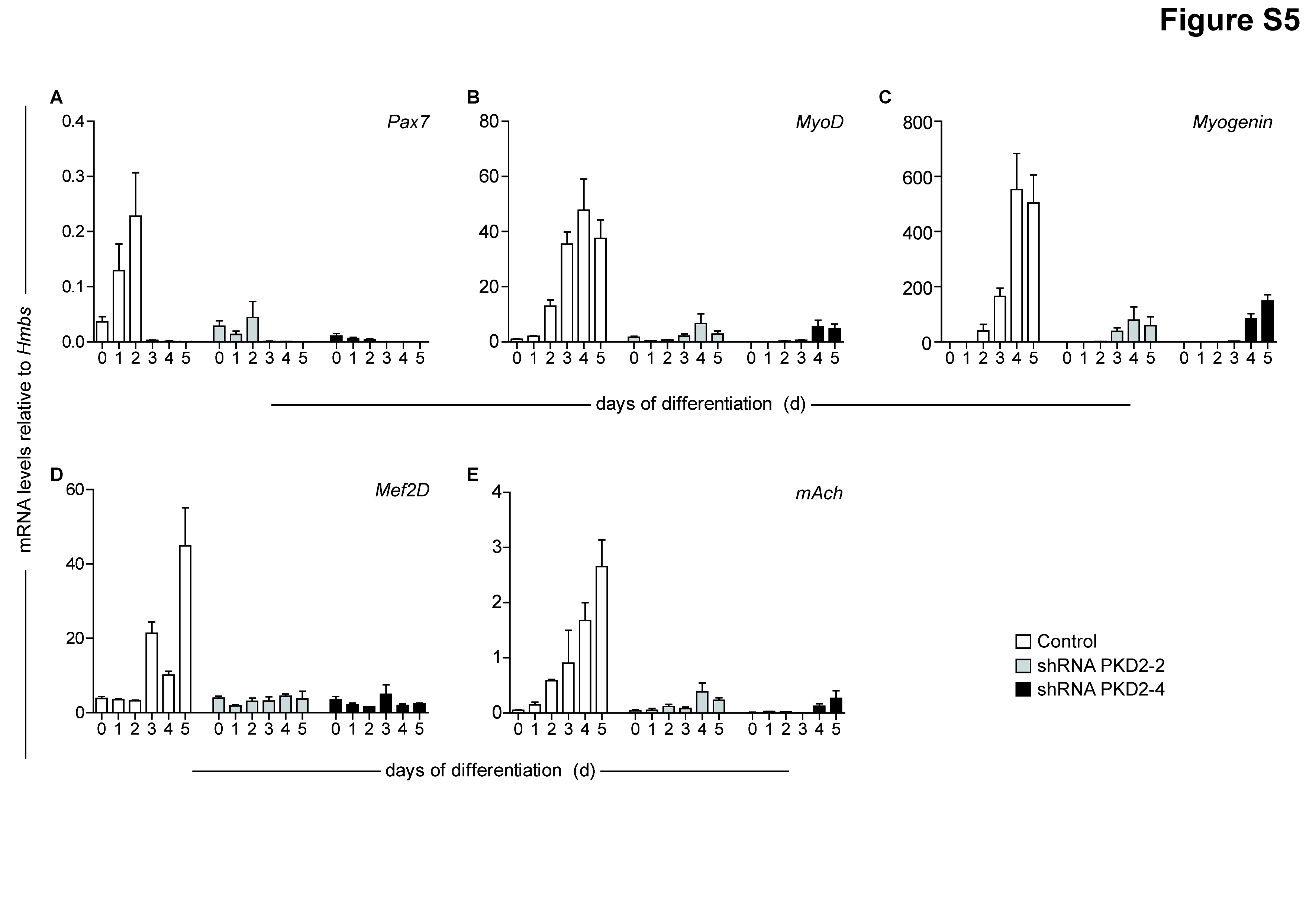

Supplement: Figure S5 — PKD2-depleted cells show altered expression of myogenic markers. Scramble-, shRNA-PKD2 construct #2 and shRNA-PKD2 construct #4 infected cells were differentiated for 5 days and mRNA samples were collected. qPCR for Pax7 (A), MyoD (B), Myogenin (C), Mef2D (D) and mAch [nicotinic, cholinergic receptor, (E)] expression (n = 3). (1.34 MB TIF) [file pone.0014599.s005.tif]

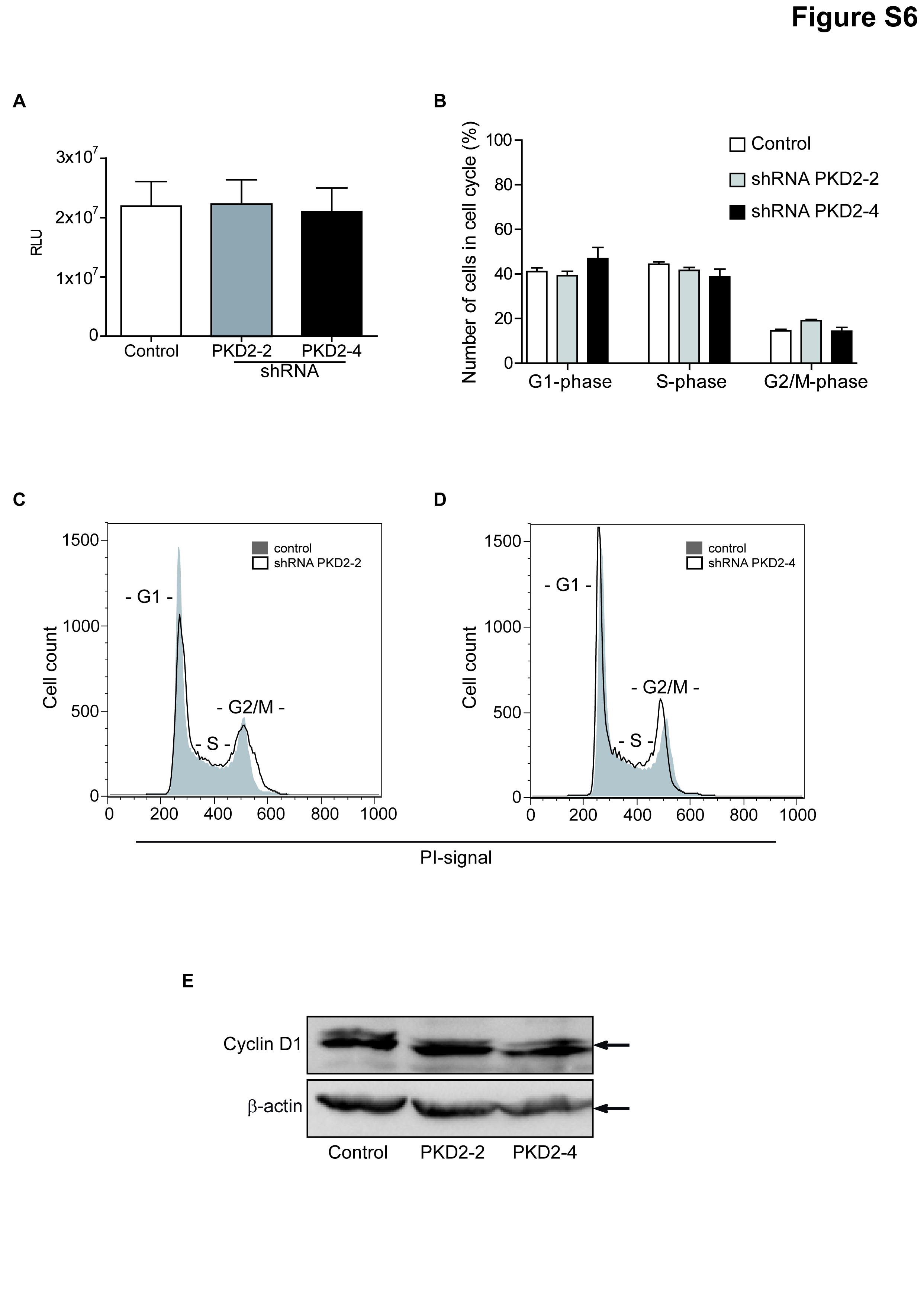

Supplement: Figure S6 — PKD2-depleted cells show normal proliferative capacity. (A) The CellTiter-Glo Luminescent Cell Viability Assay was used to determine the number of viable cells in cultures based on quantitfication of the ATP present. Values represent measurement of relative light units (RLU) 48 hours after seeding equal numbers of cells (n = 3). (B) Quantification of percentage of cells being in either G1-, S or G2/M-phase (n = 3). (C and D) Representative cell cycle plot for control vs. shRNA PKD2-2 (C) and shRNA PKD2-4 (D). (E) Immunoblot analysis for Cyclin D1 in Control- and PKD2-depleted cells after 24 hours of culture in growth medium. Representative immunoblot out of three independent experiments. (1.89 MB TIF) [file pone.0014599.s006.tif]

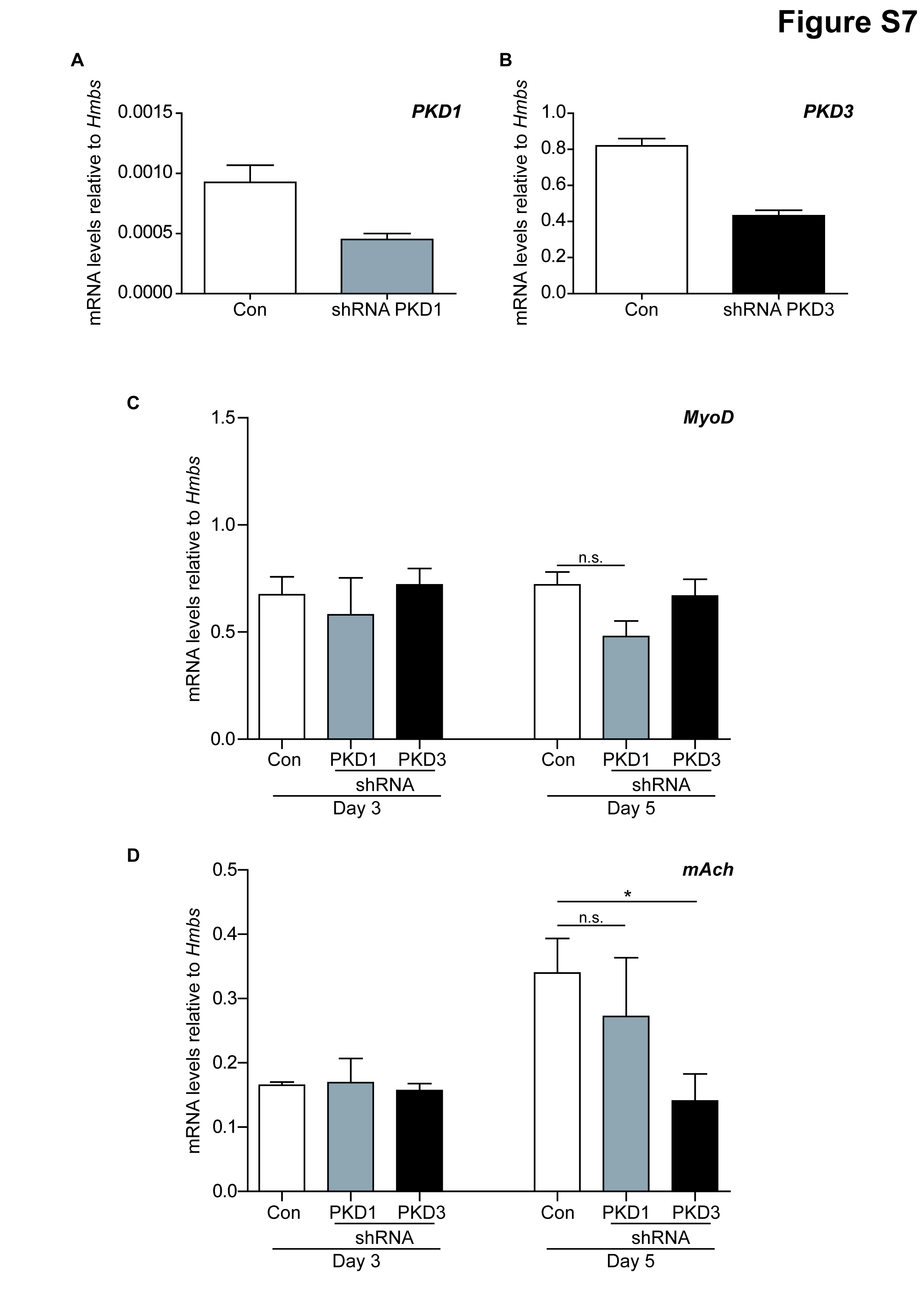

Supplement: Figure S7 — PKD1 and PKD3 are less critical for C2C12 myoblast differentiation. (A-B) Generation of stable C2C12 cell lines after lentiviral infection with either scramble shRNA (Con) or a pool of shRNAs targeting either PKD1 (A) or PKD3 (B). (C) qPCR analysis of MyoD on day 3 and day 5 of differentiation of the indicated cell lines. (D) qPCR analysis of mAch on day 3 and day 5 of differentiation of the indicated cell lines. N = 3; n.s. = not significant. (1.66 MB TIF) [file pone.0014599.s007.tif]
